# Description of the Human Penile Urethra Epithelium

**DOI:** 10.3390/medicina61050788

**Published:** 2025-04-24

**Authors:** Matisse Duval, David Brownell, Stéphane Chabaud, Alexis Laungani, Eric Philippe, Stéphane Bolduc

**Affiliations:** 1 Laboratoire d’Anatomie, Surgery Department, Faculté de Médecine, Université Laval, Quebec, QC G1V 0A6, Canada; matisse.duval.1@ulaval.ca (M.D.); eric.philippe@fmed.ulaval.ca (E.P.); 2 Centre de Recherche en Organogénèse Expérimentale/LOEX, CHU de Québec-Université Laval Research Center, Regenerative Medicine Division, Université Laval, Quebec, QC G1J 5B3, Canada; david.brownell@crchudequebec.ulaval.ca (D.B.); stephane.chabaud@crchudequebec.ulaval.ca (S.C.); 3 GrS Montréal, Montréal, QC H3L 1L2, Canada; alaungani@grsmontreal.com; 4 Division Science et Enseignement, Département de Chirurgie Plastique, Université de Montréal, Montréal, QC H3T 1J4, Canada; 5 Department of Surgery, Université Laval, Quebec, QC G1V 0A6, Canada

**Keywords:** urethra, epithelium, fossa navicularis, glycogen, gland

## Abstract

*Background and Objectives*: The male urethra is a complex structure that plays a critical role in genitourinary health and function. Despite its importance, histological descriptions of the penile urethra, particularly its epithelial components, remain incomplete. This study offers a comprehensive histological analysis of the penile urethra, focusing on the epithelium across distinct anatomical regions, including the glans, distal and proximal fossa navicularis and spongy urethra. *Materials and Methods*: Utilizing five human penile specimens, we employed various staining techniques to elucidate the structural characteristics of these epithelial tissues. *Results*: Our findings reveal notable variations in epithelial composition, such as the presence of glycogen-rich cells in the distal fossa navicularis and the presence of mucous glands in the spongy urethra and proximal fossa navicularis. Additionally, we identified a previously underreported valvule-like structure in the distal fossa navicularis in two of the specimens. In addition, the epithelium of the glans and the distal fossa navicularis are thicker than the ones of the proximal fossa navicularis and the spongy urethra. With a similar vascular density, the orientation of the blood vessels also diverges starting with the distal fossa navicularis. *Conclusions*: This study provides new insights into the histological organization of the penile urethra, offering critical reference data that can enhance our understanding of urethral pathologies and improve the outcomes of surgical interventions, particularly in the context of tissue engineering and reconstructive surgery.

## 1. Introduction

The male urethra can be susceptible to various pathologies, such as hypospadias or stenosis, which often require surgical intervention to restore function [1]. Current clinical treatments predominantly rely on the oral mucosa harvested from the inner cheek [2,3]. This tissue is advantageous due to its constant exposure to a humid environment, similar to that of the urethra. However, notable differences exist between oral and urethral mucosa, and harvesting oral mucosa can lead to complications at the donor site. Additionally, the limited quantity of tissue that can be harvested may be insufficient in some cases.

In light of these challenges, alternative sources of graftable tissues have been actively pursued, with tissue engineering emerging as a promising solution over the past few decades [4,5]. Tissue engineering enables the reconstruction of tissues in the laboratory, providing potential substitutes for organs or serving as preclinical models [6]. Regardless of the intended application, a precise understanding of the function and histology of a target tissue is essential.

Histological knowledge of the human male urethra is relatively incomplete. From the prostate to the urinary meatus, the literature generally describes two types of epithelia [7]. However, a few studies have reported additional epithelial types, particularly in specific regions such as the distal fossa navicularis. For example, Holstein et al. described a region in the distal fossa navicularis containing cells rich in glycogen, which they hypothesized may play a role in protecting against urinary tract infection [8].

To advance our understanding of human urethral histology, it is crucial to conduct a detailed study of the urethra, defining its distinct regions and transition zones. Such research is particularly important for tissue engineering, where accurately replicating the native urethra is key to ensuring the functionality of engineered substitutes. This study, based on five specimens, aims to provide valuable insights for clinicians and researchers alike, offering a comprehensive and up-to-date reference for the human urethra’s histological organization.

## 2. Materials and Methods

### 2.1. Ethics

This study was conducted according to the Declaration of Helsinki and was approved by the institution’s committee for the protection of human participants (Comité d’éthique de la recherche du CHU de Québec-Université Laval, protocol number 2012-1341). All patients provided informed written consent prior to organ collection.

### 2.2. Organ Collection and Preparation

**Figure 1:**
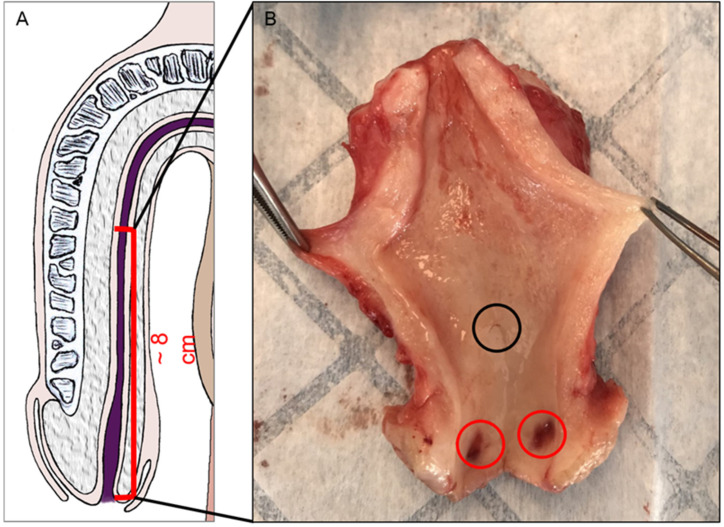
Urethra collection from male-to-female vaginoplasty surgery. A schematic representation of the region collected from the penis. (**A**) A representative photo of a specimen. (**B**) Puncture wounds are seen in the distal fossa (red circle). This specimen presented a macroscopically visible valvule (black circle).

Penis samples were collected during gender affirmation surgery in which the penis is largely removed. The informed consent of patients was obtained before samples were obtained. Five penises were collected. Prior to the operation, the patients underwent a two-year anti-androgen treatment. After removal, the penises were dissected to remove the two cavernous bodies and the skin, leaving the urethra and the glans intact. The penises were then longitudinally sectioned on the ventral side, leaving the dorsal side untouched (Figure 1). They were then stored in formol to prevent them from degrading until they were embedded in paraffin in preparation for staining. The distal part of the fossa navicularis of one of the penises was lost during preparation, reducing the sample size for several analyses to 4 instead 5 specimens.

### 2.3. Histological Studies

Four Stainings Were Applied for Each Penis.

Hematoxylin and eosin staining (H&E) uses two dyes to differentiate cellular components. The basic dye (hematoxylin) stains the nuclei of the cells. The acidic dye (eosin) stains the cytoplasm. Muscle cells and collagen fibers can also be seen in dark pink and bright pink, respectively. The slides were stained with Harris’ hematoxylin for 7 min, followed by a 5 min rinse with tap water. Alcohol acid was then applied to remove hematoxylin from non-specific structures, and tap water was used to wash away the excess. The slides were subsequently treated with a carbonate solution for one minute, followed by another rinse with tap water. Afterward, they were stained with Putt’s eosin for 3 min, rinsed with tap water, and washed three times with ethanol. Finally, the slides were treated twice with xylene and mounted.

The Laidlaw coloration was used to see the reticulin fibers, which are highlighted in black by ammoniacal silver nitrate. The slides were treated with a 0.5% periodic acid solution for 5 min, followed by a rinse with distilled water. They were then treated with 5% oxalic acid for 5 min and washed with distilled water for 10 min. Next, a solution of ammoniacal silver carbonate, heated to 58 °C, was applied to the slides for 6 min. This was followed by rinsing with bi-distilled water, water-diluted ammonium and water-diluted formalin (1%) for 5 min. The slides were then treated with 0.2% gold chloride for 10 min, which was removed by rinsing with distilled water. After being retreated in 5% oxalic acid for 5 min, the slides were rinsed with distilled water. A 5% sodium thiosulfate solution was applied for 15 min and washed off with tap water. Finally, Kernechtrot reagent (nuclear fast red stain) was applied on the slides for 5 min, followed by a rinse with tap water before mounting.

The periodic acid–Schiff (PAS) was used to highlight the glycogen in the cells of the distal fossa navicularis. The periodic acid colors the glycogen in a bright pink/purple. First, the slides were treated with a 0.5% periodic acid solution for 5 min, then rinsed with tap water. Next, they were treated with Schiff reagent for 5 min, followed by another rinse with tap water. Finally, the slides were treated with Harris’ hematoxylin to stain the nuclei, then rinsed with tap water, treated with a 1% solution of lithium carbonate for 10 s and rinsed with distilled water before the tissues were mounted on microscope slides. Since this staining technique targets carbohydrates, mucins were also stained with a similar coloration. As a negative control, an enzymatic solution containing diastase was used to digest the glycogen, which removes the bright pink/purple coloration that is normally seen in the epithelium. The sections were treated with the solution for 60 min at 37 °C before undergoing PAS staining. Since the diastase did not digest the mucins, they remained pink/purple in the PAS-digested (PASd) slides.

Alcian blue targets acidic substances. In this case, mucins were principally stained with a bright blue. Alcian blue itself is responsible for staining the mucins. The slides were treated with a 3% acetic water solution and stained with Alcian blue at 60 °C, followed by rinsing with tap water and distilled water. To counterstain, the slides were treated with Kernechtrot reagent for 6 min, then given a final rinse with distilled water before being mounted.

Representative photographs of urethra slices stained with the 4 different protocols were provided for the 4 penises in supplementary figures: hematoxylin and eosin staining (Supplementary Figure S1), Laidlaw coloration (Supplementary Figure S2), PAS (Supplementary Figure S3) and PASd (Supplementary Figure S4).

The slides were observed with a Zeiss Axioplan microscope (Carl Zeiss, Oberkochen, Germany) and scanned with a Motic EasyScan (Motic Microscopes, San Antonio, TX, USA)). ImageJ 1.53e software (NIH, Bethesda, MD, USA) was then used to create the different figures presented in this article. Since the penises were cut on the ventral side, all the observations are from the dorsal part of the penis.

### 2.4. Thickness Measurement

The thickness of the epithelium was measured on H&E-stained slices using a Zeiss Axio Imager M2 microscope equipped with an AxioCam HR Rev3 camera (Carl Zeiss, Oberkochen, Germany). Images were processed with the AxioVision 40 V4.8.2.0 software (Carl Zeiss, Oberkochen, Germany). Images were analyzed using ImageJ software: 30 measurements were made for each of the 4 distinct regions of the urethra (glans, distal and proximal fossa navicularis, spongy urethra) of the 5 penises (only 4 penises for the distal fossa navicularis).

### 2.5. Vascular Network Evaluation

The vascular network was evaluated from the H&E-stained slices using a Zeiss Axio Imager M2 microscope equipped with an AxioCam HR Rev3 camera. Images were processed with the AxioVision 40 V4.8.2. For each region (glans, distal and proximal fossa navicularis, spongy urethra), six image samples (360 µm long and 320 µm wide) were selected along the urethra just under the epithelium. Vascular structures were identified, and their surface area was measured using ImageJ. The total surface was measured and noted as the vascular density. Structures were divided into elements with a surface less than 50 µm^2^ (small elements, generally with a circular index lesser than 1.5) that represent structures transversally cut and larger elements (generally with a circular index greater than 2) that represent structures longitudinally cut. A ratio of longitudinal and transversal element total surfaces was calculated. Six measures were made for each of the 4 distinct regions of the urethra of the 5 penises (only 4 penises for the distal fossa navicularis).

### 2.6. Statistical Analyses

Graphical representation and statistical analyses were performed using GraphPad Prism 10.2.1 software. The results are expressed as mean ± standard error of the mean (SEM). Statistical analyses were performed using the two-way ANOVA followed by Tukey’s multiple comparisons tests. Statistical significance was established at *p* < 0.05.

## 3. Results

### 3.1. Epithelium of the Glans

**Figure 2:**
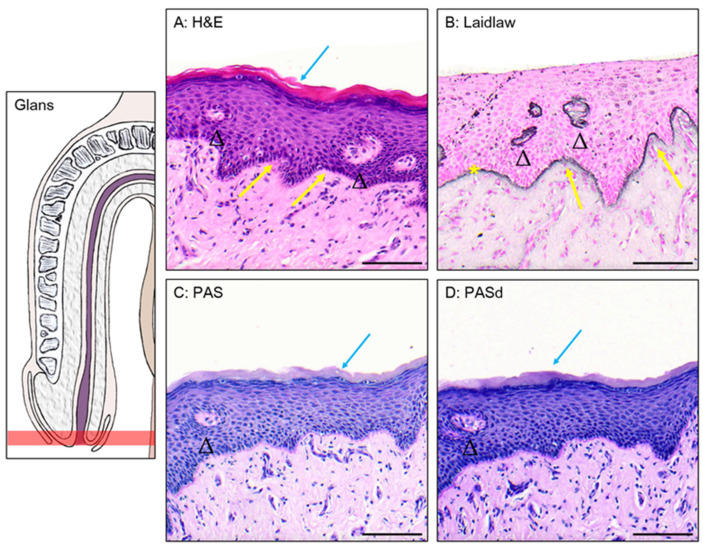
The glans urethra. H&E (**A**), Laidlaw (**B**), PAS (**C**), and PASd (**D**) staining of the glans urethra. Blue arrows highlight the keratin layer. Excretory ducts are pointed to by black triangles. Dermal papillae are labeled with yellow arrows. The basal membrane is marked by a yellow star. Scale bars represent 100 µm.

The glans is covered by a stratified squamous epithelium topped with a keratinized layer (Figure 2A). Using Laidlaw staining, the basal membrane can be seen, formed by a layer containing reticulin fibers (Figure 2B). The cells closer to the basal membrane are smaller and have a more cuboidal shape than the ones found in the middle and upper layer, which are more flattened. The cells closer to the lumen undergo deterioration to form the keratinized layer. Excretory ducts can be observed piercing through the epithelium, suggesting the presence of glands in the connective tissue. Dermal papillae, projections of the connective tissue into the epithelium, are seen at the junction between the connective tissue and the epithelium. The comparison between the PAS (Figure 2C) and PASd (Figure 2D) staining shows no presence of glycogen in this epithelium.

### 3.2. Epithelium of the Distal Fossa Navicularis (DFN)

**Figure 3:**
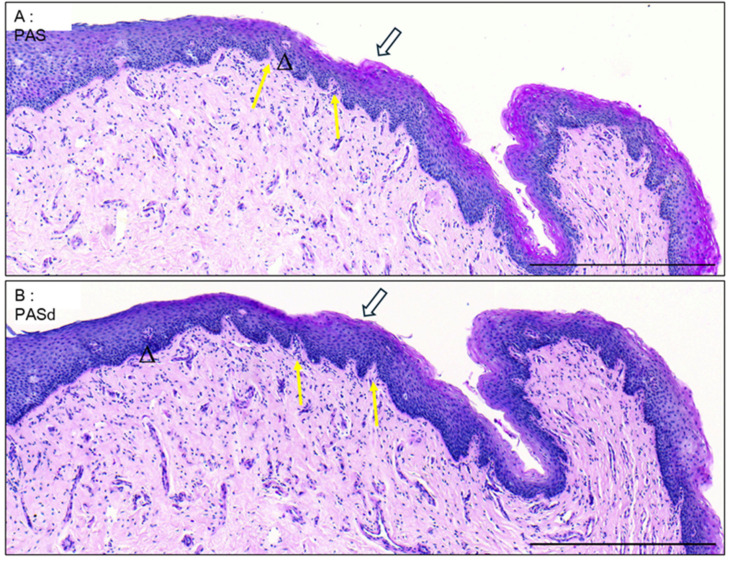
The glans-FN epithelial transition. PAS (**A**) and PASd (**B**) staining of the glans-FN epithelial transition. Glycogen apparition is pointed to by white arrows. Excretory ducts are labeled by black triangles. Dermal papillae are labelled with yellow arrows. Scale bars represent 500 µm.

**Figure 4:**
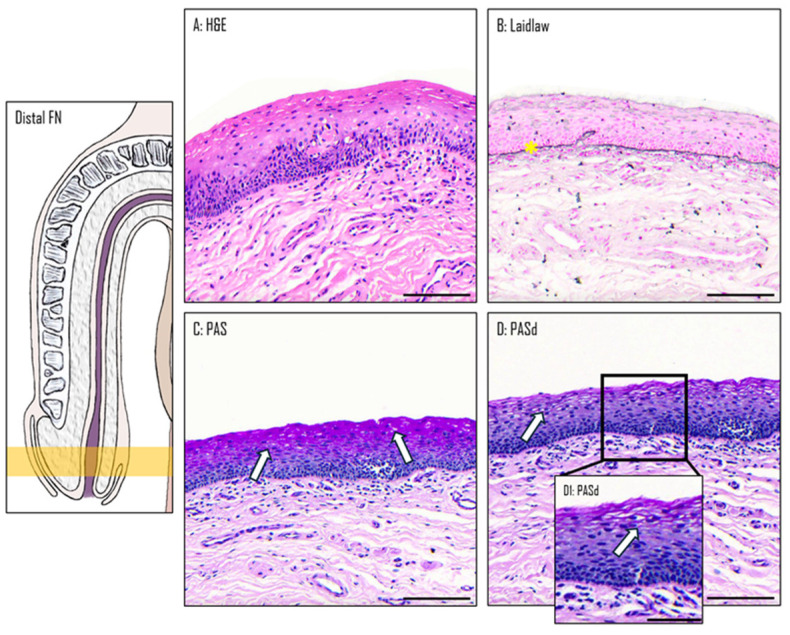
The distal fossa navicularis. H&E (**A**), Laidlaw (**B**), PAS (**C**), and PASd (**D**) staining were performed. The basal membrane is marked by a yellow star. Some glycogenated cells are labeled with white arrows. (**D1**) Shows a zoom of the epithelium. Scale bars are 100 µm for (**A**–**D**) and 50 µm for (**D1**).

Dermal papillae that are present near the transition with the glans (Figure 3A,B) are notably absent in the rest of the fossa (Figure 4A).

The DFN is covered by a stratified squamous epithelium, similar to the one of the glans (Figure 4A). However, there is no keratinized layer or excretory ducts. The basal membrane highlighted by Laidlaw staining is also observed in this part of the urethra (Figure 4B). A comparison between PAS (Figure 4C) PASd (Figure 4D) staining reveals the presence of glycogen in the upper section of the epithelium. In the three other stains, the PAS-positive cells exhibit a larger cytoplasm, allowing space for storing extra glycogen (Figure 4D, insert D1).

### 3.3. Epithelium of the Proximal Fossa Navicularis (PFN)

**Figure 5:**
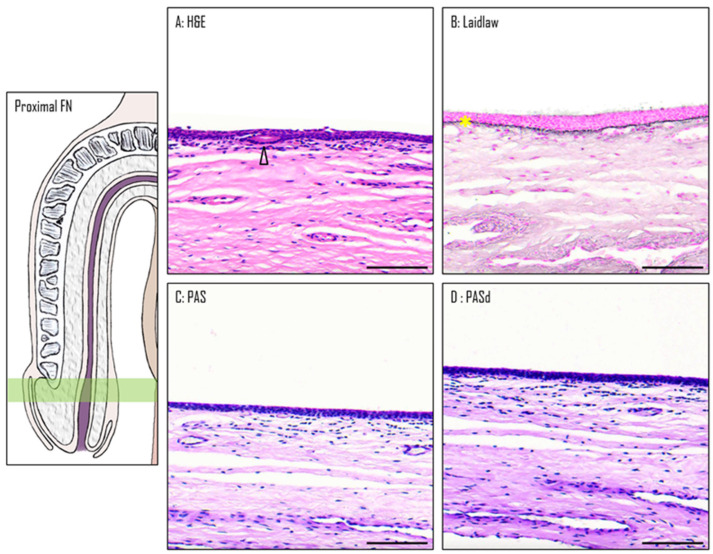
The proximal fossa navicularis. H&E (**A**), Laidlaw (**B**), PAS (**C**), and PASd (**D**) staining were performed. A gland in the epithelium is labeled with a black triangle. The basal membrane is marked with a yellow star. Scale bars represent 100 µm.

The PFN is covered by a stratified squamous epithelium, which, although similar in structure, has fewer cell layers than the ones observed in the glans and the DFN (Figure 5A). Unlike the glans and similar to the DFN, the epithelium lacks both the keratin layer and dermal papillae. One distinctive aspect of this epithelium is the presence of mucous glands and excretory ducts, which react to Alcian blue staining, suggesting an acidic secretion. As seen in the previous two sections, the basement membrane is also stained in black by Laidlaw staining (Figure 5B). The comparison between the PAS (Figure 5C) and PASd (Figure 5D) staining reveals that there is no glycogen in this epithelium.

### 3.4. Epithelium of the Spongy Urethra

**Figure 6:**
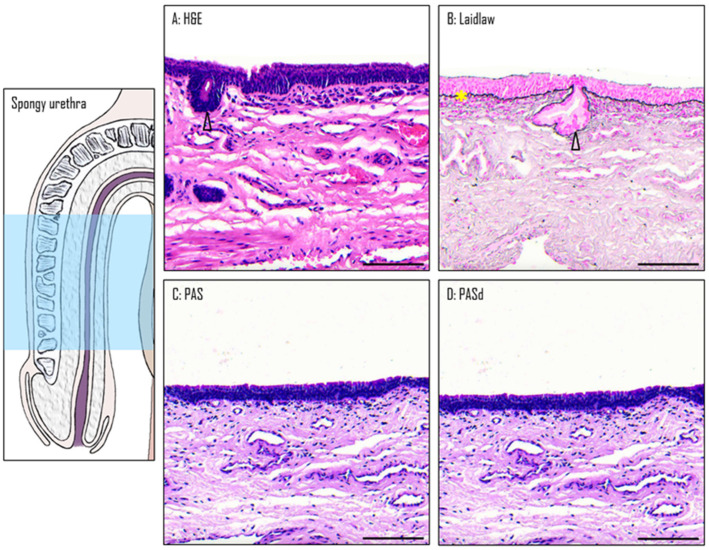
The spongy urethra. H&E (**A**), Laidlaw (**B**), PAS (**C**), and PASd (**D**) staining were performed. Glands are labeled with black triangles. The basal membrane is labeled with a yellow star. Scale bars represent 100 µm.

**Figure 7:**
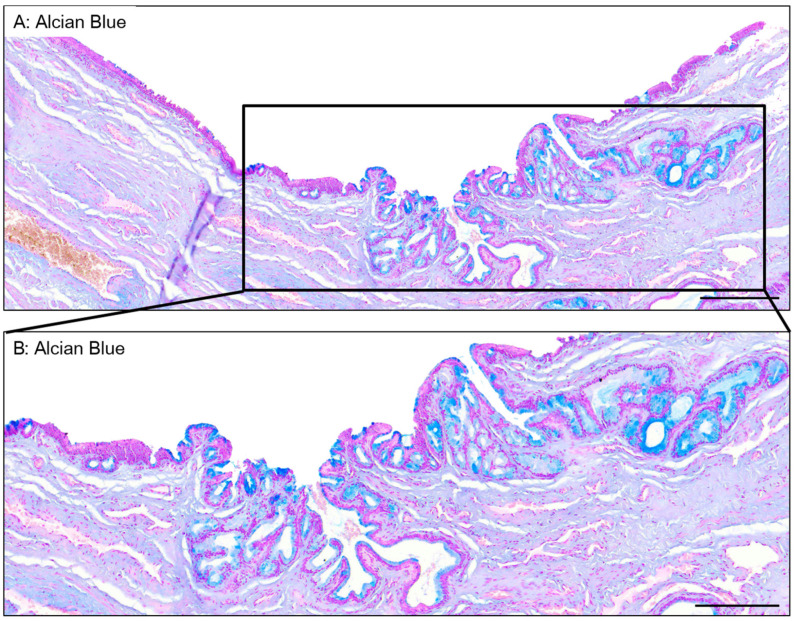
Glands in the spongy urethra. Alcian blue staining reveals large glandular structures (**A**). Details are seen in (**B**). Scale bars are 500 µm for (**A**) and 350 µm for (**B**).

The spongy urethra is lined by a pseudostratified columnar epithelium. Similarly to the one found in the PFN, it has no keratinized layer or dermal papillae (Figure 6A). Within this epithelium, mucous glands are present, similar to those found in the PFN (Figure 6B). However, the ones found in the spongy urethra are often larger in size than the ones in the PFN. They produce a mucoid substance similar to the ones found in the PFN, as the secretory cells are also stained by Alcian blue (Figure 7). The basement membrane is still visible when Laidlaw staining is applied (Figure 6B). The comparison between the PAS (Figure 6C) and PASd (Figure 6D) staining reveals once again that there is no glycogen in this part of the urethra.

### 3.5. Valvule-like Structure

**Figure 8:**
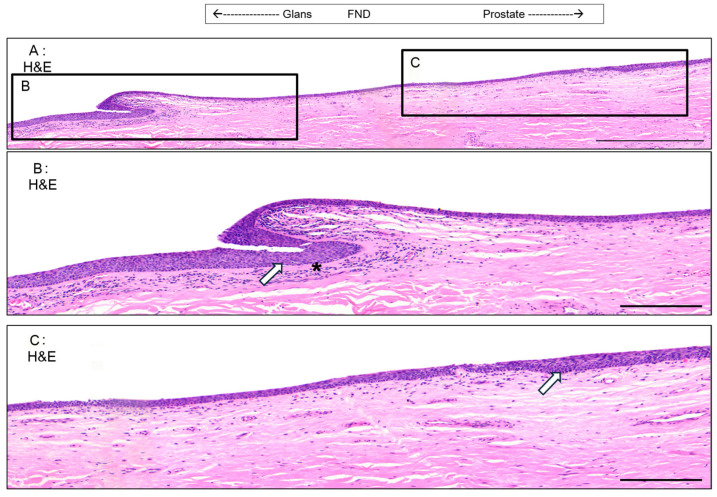
The urethral valvule. H&E staining of the distal FN (**A**). The tissue is oriented with the glans to the left and the prostate to the right. (**B**) shows a zoom of the valvule. (**C**) shows a zoom of the transition zone from a thin to a thick epithelium. High-density stromal cells are pointed out with a black star. Glycogen-containing cells are shown with a white arrow. Scale bars are 700 µm for (**A**) and 400 µm for (**B**,**C**).

In two of the examined penises, a valvule-like structure was identified in the epithelium of the DFN (Figure 8A). This structure causes a noticeable change in the epithelial thickness on either side of the valve. On the distal side (nearest to the glans), the epithelium maintains the characteristics of the DFN (Figure 8B). However, soon after the valvule and for approximately 3 mm on the proximal side of the valvule (nearest to the prostate), the epithelium becomes thinner, resembling the one found in the PFN. Beyond this 3 mm region, the epithelium returns to the typical DFN morphology, continuing until it transitions into the PFN (Figure 8C). Along with the changes in the epithelium, there are variations in the density of cells beneath the epithelium. On the distal side near the valvule, there is an increase in cell density (Figure 8B). In contrast, on the proximal side after the valve, the cell density aligns with the more typical pattern observed throughout the rest of the urethra.

### 3.6. Thickness of the Diverse Epithelia Along the Urethra

**Figure 9:**
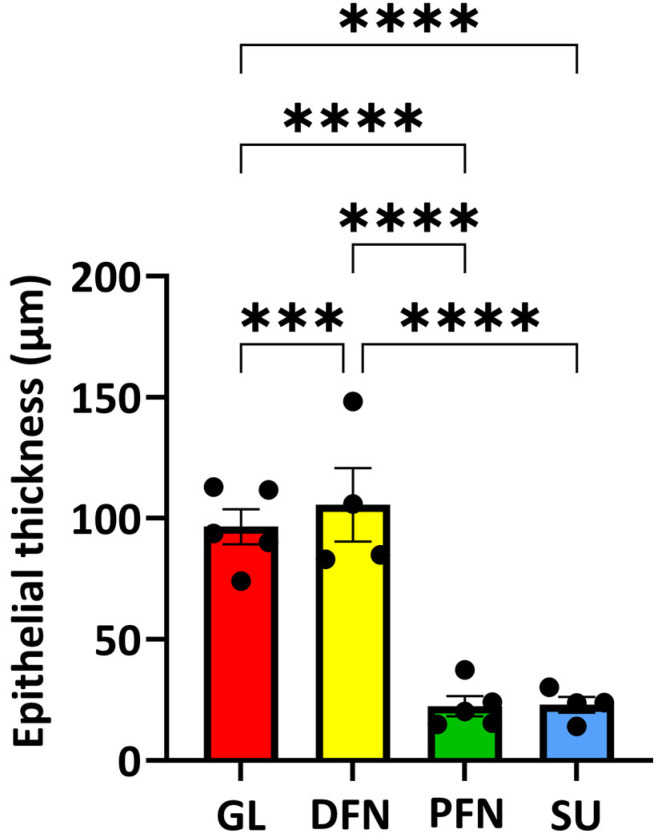
Thickness of the diverse epithelia along the urethra. The thicknesses of epithelia were measured (in µm) from the H&E-stained slices for the different areas: glans (GL, red bars), distal fossa navicularis (DFN, yellow bars), proximal fossa navicularis (PFN, green bars) and spongy urethra (SU, blue bars). Each dot represents the mean of the thirty measurements done for each patient. The data are presented as the mean +/− SEM. Statistical analyses were performed, and asterisks indicate significant differences: (***) for *p*-value < 0.001, (****) for *p*-value  <  0.0001.

The thicknesses of the epithelium were measured from the slices stained by the H&E technique. The mean +/− standard error of the mean (SEM) was determined and is presented in Figure 9. Except for the epithelia of the proximal fossa navicularis and the spongy urethra that did not show a significant statistical difference. An obvious difference distinguishes the epithelia of the glans and the distal fossa navicularis, which were the thickest, from the epithelia of the proximal fossa navicularis and the spongy urethra, which were the thinnest.

### 3.7. Organization of the Vascular Network Along the Urethra

**Figure 10:**
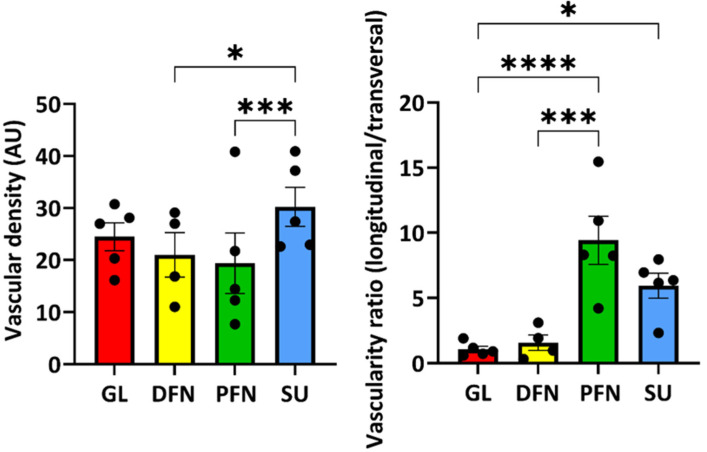
Vascular network organization. The total vascular density of the area adjacent to the epithelium (no further than 320 µm) was measured for each urethra region and plotted in arbitrary units (**left panel**), whereas the organization of this vascular network was evaluated through the surface ratio of longitudinal and transversal structures (**right panel**). Vascular densities and ratios were calculated from the H&E-stained slices for the different areas: glans (GL, red bars), distal fossa navicularis (DFN, yellow bars), proximal fossa navicularis (PFN, green bars) and spongy urethra (SU, blue bars). Each dot represents the mean of the six measurements done for each patient. The data are presented as the mean +/− SEM. Statistical analyses were performed, and asterisks indicate significant differences: (*) for *p*-value < 0.05, (***) for *p*-value < 0.001, (****) for *p*-value < 0.0001.

The vascular density of the different regions of the urethra seemed similar (Figure 10, left panel); nevertheless, the organization of the vascular network was quite different (Figure 10, right panel). The glans and distal fossa navicularis had a network organized with blood vessels in different directions (longitudinal and transversal), whereas the proximal fossa navicularis and the spongy urethra had a network mainly formed with longitudinally oriented blood vessels, i.e., parallel to the urethra lumen.

## 4. Discussion

One of the interests in making this study was to provide pictures of the different epitheliums found in the distal male urethra. Many articles describe with schematic forms what types of epitheliums are present in the urethra, but they often simplify the classification and do not provide pictures to back their observations. For example, an article published in *Nature Reviews Urology* only described two types of epithelia in the spongy urethra without any pictures [7], whereas we observed four different sections and can provide pictures to support our claims. Some articles with pictures are available, but they are rare and often old. For example, one of the only articles describing the majority of the epithelia found in the urethra [8] contains few pictures. In addition, these images were acceptable, but several factors limit the interpretations that we can make, in particular because of the absence of colors, which sometimes makes it difficult to distinguish certain structures, and the reduced variety of staining protocols used. These articles also do not provide a systematic review of all the epithelia. The authors will select specific regions of interest and ignore the transitions between the different ones.

**Figure 11:**
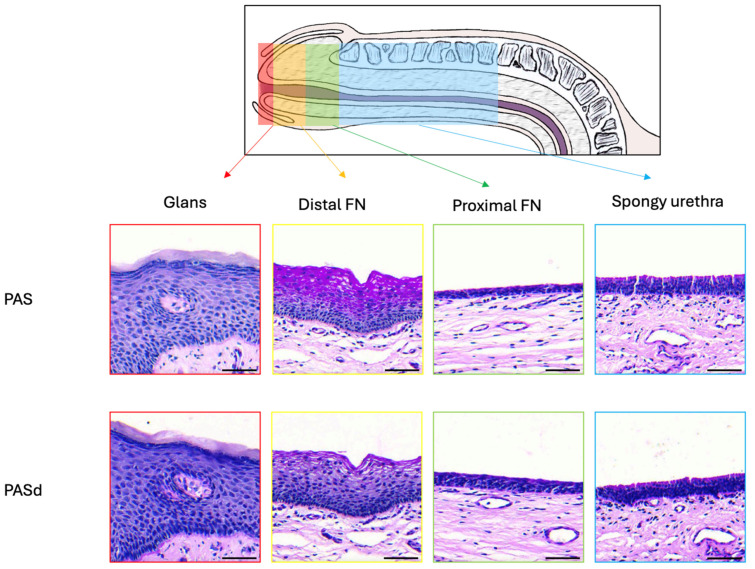
Summary of the urethral epithelial histology from the gland to the spongy urethra. Scale bars are 50 µm.

The epithelial tissues found in the distal male urethra can be categorized into four distinct types (Figure 11). The glans is lined with a stratified squamous epithelium with excretory ducts. It is the only epithelium in the urethra with dermal papillae and a keratin layer. For the distal fossa navicularis, the epithelium is also stratified and squamous but lacks the excretory ducts. Notably, this is the only epithelium in the urethra containing glycogen. Additionally, a valvule was observed in this region of the urethra in two out of the four penises examined. The proximal fossa navicularis features a smaller stratified squamous epithelium with excretory ducts and glands. Finally, the spongy urethra has a pseudostratified columnar epithelium, also containing glands and excretory ducts. The epithelial thickness and the organization of the vascular network changed drastically between the distal and proximal fossa navicularis (Figure 9 and Figure 10). Indeed, the thickness of the epithelium is greater in the glans and distal fossa navicularis compared to the proximal fossa navicularis and spongy urethra. In addition, it is obvious that despite a similar vascular density along the vicinity of the urethra, the blood vessels appear to be randomly organized in the glans and distal fossa navicularis to ensure better vascularization of these parts of the urethra, whereas the vessels in the proximal fossa navicularis and spongy urethra seem oriented in a parallel axis to the lumen of the urethra to convey the blood to the more distal regions. Once again, a clear difference appeared between the distal and proximal parts of the fossa navicularis. These quantitative observed differences correlate with a different histological organization and therefore a distinct physiological role of the four distinct regions of the urethra. This further underlines the fact that the general organization of this tissue differs markedly throughout. Those observations were consistent with all penises observed.

Our description of the epithelium lining in the glans aligns with that provided by Holstein et al. [8] but expands the findings by noting the presence of dermal papillae and excretory ducts that are in this epithelium. The distal fossa navicularis is consistent with the descriptions from both Holstein and Hausmann [9]. In the proximal portion of the fossa navicularis, our observations are similar to those of Hausmann and Holstein, although neither study reported the mucous glands we identified. The epithelium lining in the spongy urethra in our observations corresponds with descriptions found in various articles [8,10,11].

The urethra contains various epithelial structures, each serving a distinct function. The keratinized epithelium found in the glans provides better protection against friction. The dermal papillae, rich in small blood vessels, ensure adequate vascularization [12], which is essential since this epithelium has multiple cell layers. The epithelium of the distal fossa navicularis contains glycogen in its upper layers, which provides a substrate for Lactobacillus bacteria, similar to those found in the vagina. These bacteria create an acidic environment that protects the urethra from ascending pathogens [8,13]. These lactobacilli constitute the normal FN micro-biota and can have various roles as demonstrated in FN or vagina: modulating the inflammatory response [14,15], promoting wound closure [15] and protecting against infections [16]. Protection from microbial, bacterial or viral infections is primarily due to the mucus, which traps microbes, but also to the acidification of the environment through the production of lactic acid [17,18]. There is also production of hydrogen peroxide and bacteriocins [19,20]. Finally, this flora competes directly with pathogens for their adhesion sites. Faster wound healing also limits the entry of pathogens. A decrease in lactobacilli in the FN or vagina correlates with an increase in sexually transmitted infections [16,21,22,23,24,25]. The epithelium in the spongy urethra, which expands through most of the urethra [1], forms an impermeable route for the urine and the sperm to exit the body, while protecting the underlying tissue [26].

These diverse epithelial structures found in the urethra highlight the importance of integrating the multiple types of epitheliums observed when reconstructing a urethra for patients with hypospadias. While the urothelium provides an impermeable pathway for the urine and the sperm, the epithelium of the distal fossa navicularis offers protection against infections. Current gold-standard tissue engineering techniques for urethral reconstruction only use the urothelium [7], but this approach does not utilize the protection offered by the fossa navicularis.

Currently, urethral reconstruction mainly relies on the use of autologous oral mucosa. This process can be painful for the patient and may cause side effects at the donor site. In addition, the nature of the oral epithelium is different from that of the epithelium of urological tissues, especially in terms of permeability. These differences can be responsible for subsequent inflammation, fibrosis, and stenotic recurrence. In addition, the anatomical harvesting site does not always allow a sufficient surface area of tissue to carry out the most complex repairs and is unavailable for subsequent surgeries in case of recurrences [1]. Many teams have undertaken the reconstruction of urethral tissues using tissue engineering. Most of the proposed techniques are based on the use of biomaterials (such as poly-lactic acid (PLA) and poly-glycolic acid (PGA), poly(lactic-co-glycolic) acid (PLGA), poly(ε-caprolactone) (PCL), polycaprolactone/poly(L-lactide-co-ε-caprolactone) (PLCL)) to build a scaffold and this with different approaches (such as casting, electrospinning, printing) [1,27]. Some groups have also suggested the use of acellularized tissues (small intestinal submucosa (SIS), and bladder acellular matrix (BAM) [1,28,29,30]. The choice of cells to populate the scaffolds is also essential [31,32] In most cases, the cells used to populate the grafts are not of urothelial origin, leading to inadequate cellular differentiation. Often, the urothelia only have one to three layers of cells, and the presence of mature uroplakin plaques is very rare even though these are essential to limit urine leakage, which leads to fibrosis and recurrences. None of the techniques used has demonstrated extensive clinical use over time and in sufficient numbers. More recently, research has been undertaken to reconstruct tissues without exogenous biomaterials and using cells specific to urological tissues [33]. This work allows the production of flat or tubular urethral substitutes, showing a unique level of differentiation of epithelia with adequate functional properties (including permeability and mechanical properties). These tissues can be endothelialized and show faster reperfusion once grafted (4 days instead of 10) [34]. In addition, these kinds of tissues can be matured in a bioreactor to decrease the permeability and improve the mechanical properties [6]. The first assays on animals have been carried out successfully and are to be expanded soon. However, since the current gold standard for tissue engineering for urethral reconstruction only uses a urothelium [7], the tissue is uniform and does not have successions of regions as described in this article. This prevents the protection against infections provided by the fossa navicularis. These diverse epithelial structures found in the urethra highlight the importance of integrating the multiple types of epitheliums observed when reconstructing a urethra for patients with hypospadias. It is necessary to note that such a re-construction process can also be useful in the case of reparation of urethral stenoses close to the glans. Despite the complexity of such an approach, it seems obvious that the presence of several regions presenting histological and functional differences obliges us to change our reconstruction strategies now to best serve patients.

Along the urethra, many glands can be found, either in the epithelium or in the stroma. They are mostly present in the distal part of the spongy urethra and at the proximal end of the proximal fossa navicularis. These glands correspond to Littré’s glands [35]. Two types were described by Krstić. The ones present in the epithelium are small and contain mucus-secreting cells. The ones in the connective tissue are bigger and have excretory ducts opening into the epithelium [11]. We observed both types in our penises. The mucus secreted by these glands contains glycosaminoglycans, which protect the epithelium from the urine and pathogens and lubricate the urethra to allow better passage for the urine and sperm [11,36,37].

Beneath the epithelium lies a dense network of blood vessels, which ensures the efficient distribution of nutrients. Since there is no vascularization in the epithelium, the cells must get their nutrients and eliminate their waste through diffusion, explaining the need for intense vascularization under the basal membrane. In this study, the identification of vascular structures relied on histological features. In the future, molecular markers such as PECAM-1 or von Willebrand factor [34] could be used to detect the network of blood vessels/capillaries. Pancytokeratins or K5 +/− p63, more specific to urothelial stem cells [31,38], could be used to identify epithelial cells.

The two valvules seen in the distal fossa navicularis have previously been described as the lacuna magna or sinus of Guérin [39]. This lacuna is formed by a septum (valve of Guérin), an extension of the ingrowth of the ectoderm that would normally form the distal fossa navicularis [40]. This lacuna may also cause different symptoms, such as dysuria and post-void bloody spotting [41]. Shenoy also describes the valve of Guérin as a “horizontal bar stretched across the roof of the navicular fossa” and documented observing it in 98% of cases. A reason explaining why we only found this valve in 50% of our penises comes from its small size and its localization varying from one person to another [42]. It is then possible that depending on the site of sampling for histology, the valvule is not present, or it is also possible that the size of the valvule can differ from patient to patient and can be ignored by some histologists.

A limitation in this study comes from the fact that all the patients underwent a minimum of two years of hormonal treatment prior to gender affirmation surgery. Given that estrogen treatments can cause expansion of the distal fossa navicularis [8], it is possible that this area expanded, altering the organization of the urethra. However, this expansion could be advantageous for these patients. Specifically, if cells from the distal fossa are extracted and harvested, they could be used in tissue engineering to create vaginal-like tissue, which could then be grafted during the surgery. This approach may offer a better alternative to the current gold standard in vaginoplasty, which is penile inversion [42]. Penile inversion has several downsides, including limited tissue availability and the absence of protective vaginal flora. At this moment, the only tissue engineering methods available for vaginoplasty use buccal mucosa cells [43]. However, this technique takes time and can create morbidity at the donor site [44]. Using the patient’s distal fossa navicularis cells to generate tissue could provide better protection against pathogens and HIV, as glycogen is present in the epithelium [13]. Comorbidities could also be reduced [45], which would further enhance patient outcomes.

## 5. Conclusions

To conclude, the epithelium of the penile urethra is composed of at least four distinct zones, which present a different histological organization adapted to physiological functions. The epithelium of the fossa navicularis shows a histology close to that of the vagina with the function of serving as a gatekeeper to prevent the entry of bacteria. The reconstruction of such an area could be considered during reconstruction by tissue engineering, particularly in the case of treatment of hypospadias, in order to limit potential bacterial infection.

## Data Availability

Data are available upon reasonable request.

## Supplementary Materials

The following supporting information can be downloaded at https://www.mdpi.com/article/10.3390/medicina61050788/s1: Figure S1: Representative photographs of hematoxylin and eosin staining of the urethra; Figure S2: Representative photographs of Laidlaw coloration of the urethra; Figure S3: Representative photographs of the periodic acid–Schiff staining of the urethra; Figure S4: Representative photographs of the periodic acid–Schiff staining of the urethra after diastase digestion.
