# Supplementary material for: Description of the Human Penile Urethra Epithelium
Source: Medicina (Kaunas). 2025 Apr 24;61(5):788. doi: 10.3390/medicina61050788 (PMC12113345; doi:10.3390/medicina61050788)
Supplement: Supplementary file 1 [file medicina-61-00788-s001.zip › medicina-3545254-supplementary.pdf]

Supplementary data

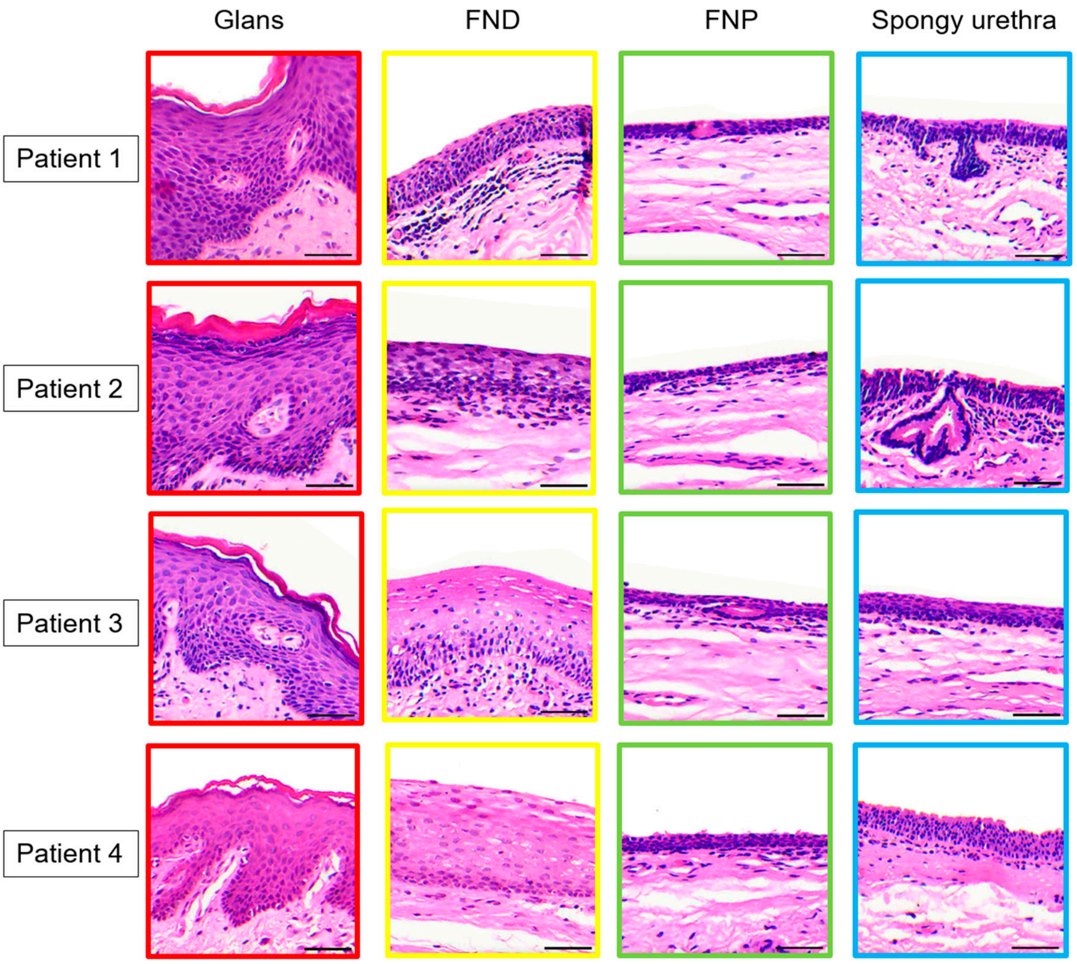

Supplementary figure S1: Representative photographs of Hematoxylin-Eosin staining of the urethra. Tissue slices were stain from 4 regions of the urethra, the glans (in red), the distal Fossa Navicularis (FND, in yellow), the proximal Fossa Navicularis (FNP, in green) and the spongy urethra (in blue) for 4 donor patients. Scale bars represent 100 µm.

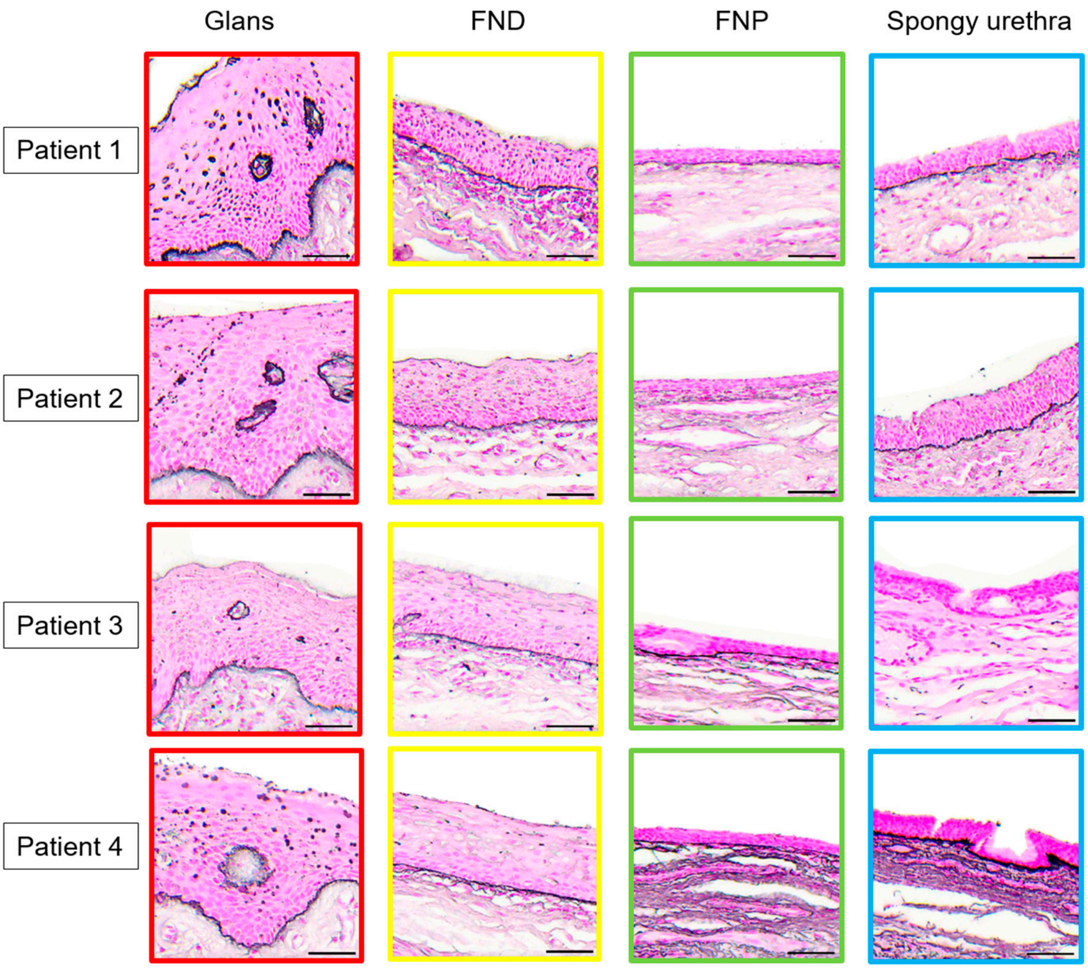

Supplementary figure S2: Representative photographs of Laidlaw coloration of the urethra. Tissue slices were stain from 4 regions of the urethra, the glans (in red), the distal Fossa Navicularis (FND, in yellow), the proximal Fossa Navicularis (FNP, in green) and the spongy urethra (in blue) for 4 donor patients. Scale bars represent 100 µm.

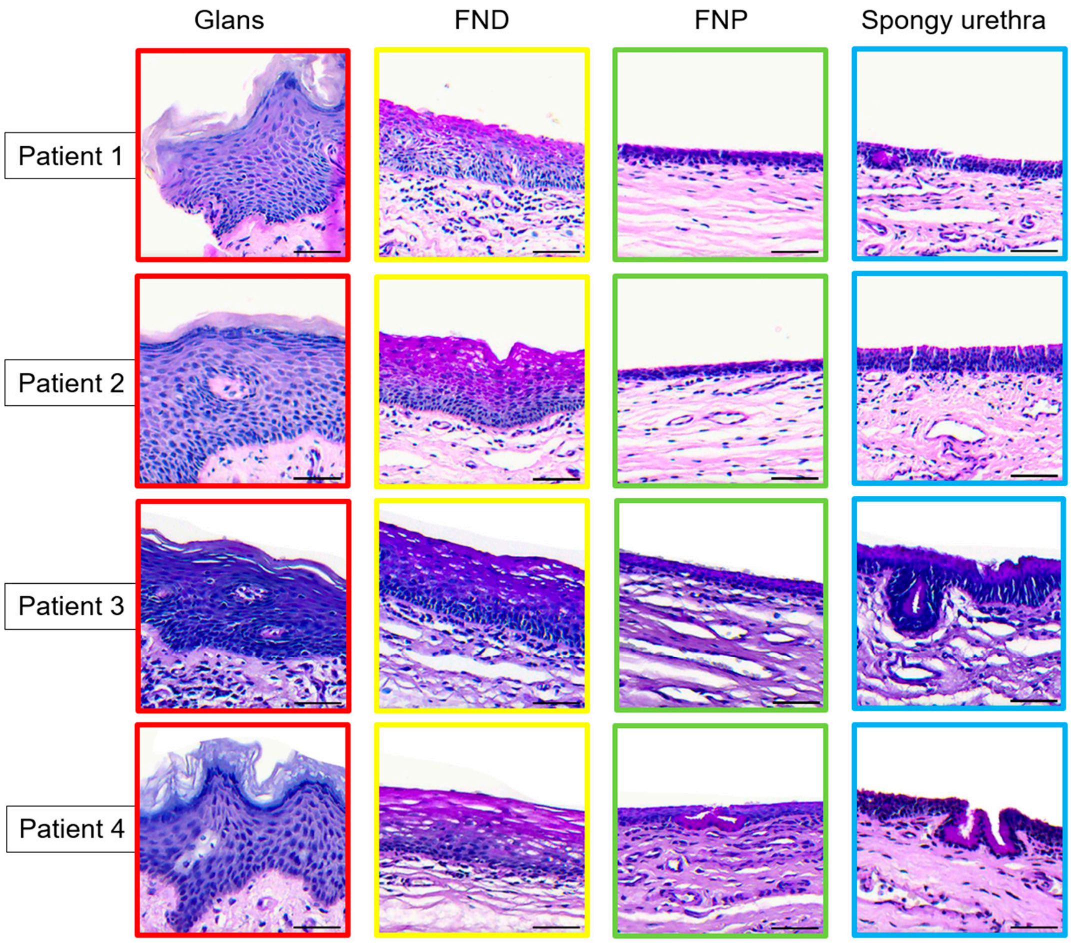

Supplementary figure S3: Representative photographs of the Periodic-Acid Schiff staining of the urethra. Tissue slices were stain from 4 regions of the urethra, the glans (in red), the distal Fossa Navicularis (FND, in yellow), the proximal Fossa Navicularis (FNP, in green) and the spongy urethra (in blue) for 4 donor patients. Scale bars represent 100 µm.

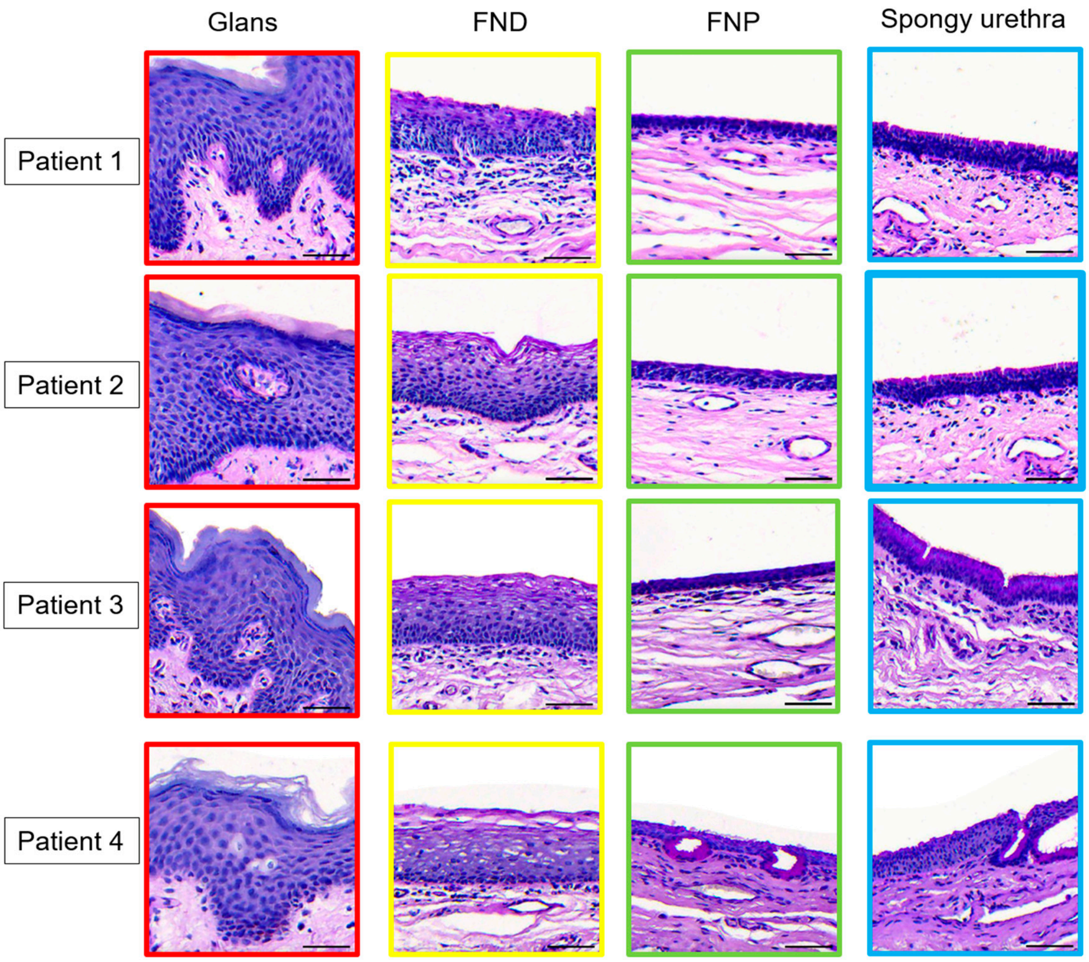

Supplementary figure S4: Representative photographs of the Periodic-Acid Schiff staining of the urethra after diastase digestion. Tissue slices were stain from 4 regions of the urethra, the glans (in red), the distal Fossa Navicularis (FND, in yellow), the proximal Fossa Navicularis (FNP, in green) and the spongy urethra (in blue) for 4 donor patients. Scale bars represent 100 µm.
